# Molecular investigation of *Cryptosporidium* and *Giardia* in pre- and post-weaned calves in Hubei Province, China

**DOI:** 10.1186/s13071-017-2463-3

**Published:** 2017-10-25

**Authors:** Yingying Fan, Tao Wang, Anson V. Koehler, Min Hu, Robin B. Gasser

**Affiliations:** 1 State Key Laboratory of Agricultural Microbiology, College of Veterinary Medicine, Huazhong Agricultural University, Wuhan, Hubei 430070 China; 2 Department of Veterinary Biosciences, Melbourne Veterinary School, The University of Melbourne, Parkville, VIC Australia

**Keywords:** *Cryptosporidium*, *Giardia*, Calves, PCR-based sequencing, Nuclear ribosomal RNA genes, China

## Abstract

**Background:**

The protistan pathogens *Cryptosporidium* and *Giardia* can cause significant intestinal diseases in animals and humans. Cattle, particularly calves, carrying these protists can be significant reservoirs for human infections and disease. However, little is known about the genetic make-up of *Cryptosporidium* and *Giardia* populations in cattle and other ruminants in some regions of China.

**Results:**

In the present study, PCR-based tools were used to genetically characterise these protists in faecal samples from a total of 339 pre- and post-weaned calves from four distinct locations in Hubei Province using markers in the large (*LSU*) or small (*SSU*) subunits of nuclear ribosomal RNA genes. *Cryptosporidium andersoni*, *C. bovis*, *C. ryanae* and *Giardia duodenalis* assemblage E were detected in 0.6%, 10.9%, 4.1% and 22.6% of calves, respectively.

**Conclusions:**

This study is the first to report the prevalence of *Cryptosporidium* and *Giardia* in pre- and post-weaned calves in Hubei Province, and encourages large-scale molecular studies of animals and humans, in an effort to better understand the epidemiology of these enteric pathogens in China.

**Electronic supplementary material:**

The online version of this article (10.1186/s13071-017-2463-3) contains supplementary material, which is available to authorized users.

## Background

*Cryptosporidium* and *Giardia* are protistan pathogens that can cause intestinal diseases in animals and humans. Using molecular tools, numerous species/genotypes of *Cryptosporidium* [1] or species/assemblages of *Giardia* have been reported to infect humans [2]. However, based on current molecular data, *Cryptosporidium hominis* and *C. parvum* as well as *Giardia duodenalis* (syn. *Giardia intestinalis*, *Giardia lamblia*) assemblages A and B are responsible for most (> 99%) human infections [3, 4]. Of these parasites, *C. hominis* infection is considered to be acquired by anthroponotic transmission [5], whereas *C. parvum* and *G. duodenalis* (mainly assemblages A and B) can be transmitted anthroponotically or zoonotically [1, 3, 4].

Cattle, particularly calves, infected with these protists can represent a significant source of zoonotic infections and disease [4, 6]. Nevertheless, cattle can become infected with various species of *Cryptosporidium* or *Giardia* [7, 8]. The species and genotypes or assemblages of these protists are known to vary among hosts of different age groups [4, 8] and geographical locations [9, 10], and new, previously undescribed genotypes of *Cryptosporidium* are being discovered in regions of the world not explored previously (cf. [11, 12]). Therefore, establishing and comparing the specific and/or genotypic identity of these protists in animals and humans in geographical regions in which there is limited molecular epidemiological information are central to assessing their zoonotic potential.

In China, the diseases caused by *Cryptosporidium* and *Giardia* are not notifiable, but infected cattle are believed to serve as significant reservoirs for zoonotic infections [13]. Although there is a positive association between the density of domestic livestock, including cattle, and the prevalence of *Cryptosporidium* infection in humans in other countries (e.g. New Zealand) [14], there are presently relatively limited epidemiological data in some parts of China to support this observation. Most previous studies describing the genetic characterization of *Cryptosporidium* from calves have been conducted mainly in the northeast, northwest and central regions of China [13, 15–22]. Similarly, limited molecular data are available for *Giardia* of cattle in other regions, although *G. duodenalis* assemblages A and B have been identified in calves [16, 17, 23–26]. In Hubei Province, the only studies of *Cryptosporidium* and *Giardia* are those in diarrhoeic children [27] and goats [28].

The aim of the present study was to explore the species and genotypes of *Cryptosporidium* and assemblages of *Giardia* in calves on farms in Hubei Province, China, using markers in the large (*LSU*) or small (*SSU*) subunits of nuclear ribosomal RNA genes, and to assess the zoonotic potential of these parasites.

## Methods

**Fig. 1:**
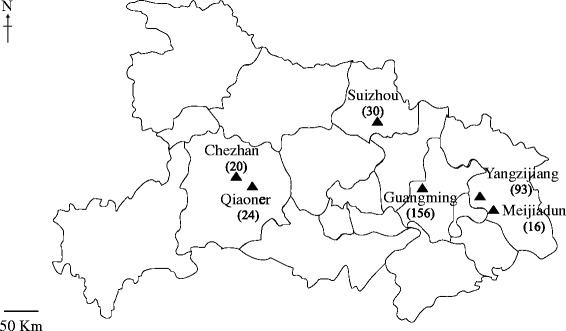
Locations of the six farms in Hubei Province from which faecal samples (numbers in parentheses) were collected from pre- and post-weaned calves and tested using PCR-based methods

A total of 339 fresh faecal samples were collected (September to December 2016) from pre- and post-weaned calves (1 to 12 weeks old) from one beef farm (i.e. Suizhou) and five dairy farms (i.e. Chezhan, Guangming, Meijiadun, Qiaoner and Yangzijiang) in the north, east and west regions of Hubei Province, China (Fig. 1). Faecal samples were collected rectally from individual calves and kept at 4 °C following sampling, and then frozen at −20 °C for subsequent DNA isolation and molecular testing. Genomic DNA was extracted from 0.2 g of each faecal sample using the PowerSoil DNA Isolation Kit (MoBio, Carlsbad, USA), according to the manufacturer’s protocol, and stored at −20 °C. This kit was used, as it is highly effective at removing components that are inhibitory to PCR [29–31]. Aliquots (2 μl) of individual genomic DNA samples were subjected to nested PCR-based amplification and sequencing, employing (individually) two distinct loci of nuclear ribosomal DNA in separate assays. For detection of *Cryptosporidium*, a portion of the large subunit of the nuclear ribosomal RNA gene (p*LSUc*; ~500 bp) was used [32], and further genotypic/subgenotypic classification was achieved by employing a portion of the small subunit of the nuclear ribosomal RNA gene (p*SSUc*; ~ 590 bp) [33]. For the specific and assemblage-based classification of *Giardia*, a portion of the *SSU* gene (p*SSUg*; ~ 290 bp) was employed [34, 35] (Additional file 1: Table S1).

In brief, nested PCRs were carried out in 50 μl using a standard reaction buffer, 2.0–3.0 mM of MgCl_2_ (depending on the locus), 200 μM of each dNTP, 50 pmol of each primer and 1 U of *Taq* polymerase (Mango DNA polymerase, Bioline, London, UK) using established cycling protocols (Additional file 1: Table S1). Except for the no-template controls, 2 μl of genomic DNA were added to the primary PCR, from which 1 μl was carried over to the secondary PCR. Known test-positive, test-negative and no-template controls were included in each PCR run.

All nested PCR products were detected by electrophoresis in ethidium bromide-stained (1.5%) agarose gels before sequencing. For sequencing, aliquots (5 μl) of individual amplicons (undigested) were treated with the enzymes *Exo* I and a thermosensitive alkaline phosphatase (FastAP, Thermofisher, Carlsbad, USA), according to the manufacturer’s instructions, and then subjected to direct, automated sequencing (BigDye Terminator v.3.1 chemistry, Applied Biosystems, USA) in both directions using the same, internal primers as employed in PCR. The quality of each sequence was assessed based on the corresponding chromatogram, and sequences were matched to reference sequences from the GenBank database (listed in Additional file 2: Table S2) using the Basic Local Alignment Search Tool (BLAST; http://www.ncbi.nlm.nih.gov/BLAST). Chi-square test was performed using SPSS Statistics 24 software (IBM, New York, USA).

## Results and discussion

Using two separate PCR assays, the 339 individual faecal DNA samples from six farms from four counties/cities of Hubei Province were screened by PCR for the presence of *Cryptosporidium* spp. and *Giardia duodenalis* DNA, respectively. In total, 15.5% (*n* = 53) and 22.6% (*n* = 77) faecal DNA samples were test-positive for *Cryptosporidium* and *Giardia*, respectively. Amplicons were sequenced, and the nucleotide sequences of *Cryptosporidium* and *Giardia* were deposited in the GenBank database under accession nos. MF196907–MF196910.

### *Cryptosporidium*

Nested PCR-based sequencing of p*LSUc* identified 53 samples that were test-positive for three *Cryptosporidium* species, including *Cryptosporidium andersoni* (0.6%; 2 of 339) on two farms, and *C. bovis* (10.9%; 37 of 339) and *C. ryanae* (4.1%; 14 of 339) on five farms (Table 1). No mixed infections of these species were detected. Farm Meijiadun showed the highest overall infection rate (31.3%; 5 of 16) of *Cryptosporidium*, whereas farm Suizhou had the lowest rate (3.3%; 1 of 30). At least one *Cryptosporidium* species was detected on each farm. *Cryptosporidium andersoni* was found only on farms Qiaoner and Yangzijiang in two pre-weaned calves of 2 and 8 weeks of age, respectively. *Cryptosporidium bovis* was the major species on farms Yangzijiang, Meijiadun, Qiaoner and Guangming, whereas *C. ryanae* was the dominant species on farms Chenzhan and Shuizhou (Table 1). There was no significant difference in *Cryptosporidium* prevalence between the beef farm and the dairy farms (*χ* ^2^ = 3.776, *df* = 1, *P* = 0.0520).

**Table 1:** Occurrence of *Cryptosporidium andersoni*, *Cryptosporidium bovis*, *Cryptosporidium ryanae* and *Giardia duodenalis* in faecal samples from calves

Farm	No. of samples tested	No. of samples test-positive for *Cryptosporidium* spp. (%)	*Cryptosporidium* species			No. of samples test-positive for *Giardia duodenalis* (%)
			*C. andersoni* (%)	*C. bovis* (%)	*C. ryanae* (%)	
Suizhou (beef farm)	30	1 (3.3)	0	0	1 (3.3)	7 (23.3)
Chezhan (dairy farm)	20	5 (25.0)	0	1 (5.0)	4 (20.0)	12 (60.0)
Guangming (dairy farm)	156	21 (13.4)	0	17 (10.9)	4 (2.6)	31 (19.9)
Meijiadun (dairy farm)	16	5 (31.2)	0	5 (31.3)	0	3 (18.8)
Qiaoner (dairy farm)	24	8 (33.3)	1 (4.2)	4 (16.7)	3 (12.5)	15 (57.7)
Yanzijiang (dairy farm)	93	13 (14.0)	1 (1.1)	10 (10.8)	2 (2.2)	9 (9.7)
Total	339	53 (15.6)	2 (0.6)	37 (10.9)	14 (4.1)	77 (22.6)

Further analysis of p*SSUc* nucleotide sequence data showed that all sequences obtained were identical to reference sequences from GenBank (Additional file 2: Table S2). For *C. andersoni*, the sequence matched that recorded previously in Chongqing, China (calves; JX515549; unpublished); for *C. bovis*, three sequences matched those recorded in Qinghai Province (yak; KU052813; [36]), Sichuan Province (cattle; KT884495; unpublished) or Gansu Province (cattle; KP994913; [37]), China; for *C. ryanae*, two sequences matched those recorded previously in Gansu Province (cattle; KP994915; [37]) and Xinjiang Province (cattle; KP793013; [13]), China.

*Cryptosporidium* was found across both genders and all age groups of calves (Table 2). The results show that 8.2% (4 of 49) of male and 16.9% (49 of 290) of female calves were test-positive for *Cryptosporidium*, whereas 15.8% (42 of 265) of pre-weaned calves (≤ 8 weeks) and 15.2% (11 of 74) of post-weaned calves (> 8 weeks) were identified as test-positive using the PCR-coupled sequencing approach for p*SSUc* (Table 2). The Chi-square test did not show any significant age- or gender-associated difference in the prevalence of *Cryptosporidium* infection (*P* = 0.838 and 0.119, respectively).

**Table 2:** Age and gender distributions of *Cryptosporidium* spp. and *Giardia duodenalis* detected

Groups	No. of samples tested	No. test-positive for *Cryptosporidium* spp. (%)	*Cryptosporidium* spp.			No. test-positive for *Giardia duodenalis* (%)
			*C. andersoni* (%)	*C. bovis* (%)	*C. ryanae* (%)	
Age (weeks)						
Pre-weaned (≤ 8)	265	42 (15.8)	2 (0.8)	32 (12.1)	8 (3.0)	49 (18.5)
Post-weaned (> 8)	74	11 (14.9)	0	5 (6.8)	6 (8.1)	28 (37.8)
Gender						
Female	290	49 (16.9)	2 (0.7)	34 (11.7)	13 (4.5)	66 (22.8)
Male	49	4 (8.2)	0	3 (6.1)	1 (2.0)	11 (22.4)

In China, increased attention is now being paid to cryptosporidiosis of livestock [38] due its clinical significance in young farm animals [39, 40] and potential to spread to humans. Previous studies have indicated that *Cryptosporidium* has a relatively wide distribution in cattle of different breeds and ages in China (reviewed by [38]). Since the initial detection of *Cryptosporidium* in diarrhoeic calves (prevalence: 45.2%; 19 of 42) in Gansu Province [41], at least nine species of *Cryptosporidium* (i.e. *C. andersoni* [21], *C. bovis* [21], *C. parvum* [15], *C. ryanae* [18], *C. ubiquitum* [42], *C. meleagridis* [22], *C. xiaoi* [42], *C. serpentis* [43] and *C. suis-like* [44]) have been reported to date in cattle in 19 regions/provinces of China. Of these nine *Cryptosporidium* species, *C. andersoni*, *C. bovis*, *C. parvum* and *C. ryanae* were the predominant species in cattle in China [38]. Although *C. parvum* has been described previously as being more common in pre-weaned calves in numerous industrialised countries (e.g. Australia, Belgium, Japan and the USA [45–48] as well as some provinces/regions of China [13, 15–17]), this was not the case in the present study, in which *C. bovis* was identified as being the predominant species in pre-weaned calves in Hubei Province. This finding is consistent with previous observations in the provinces Gansu [37], Henan [21], Helongjiang [22], Shaanxi [19] as well as in Shanghai [49]. Interestingly, the predominance of *C. bovis* in pre-weaned calves in China contrasts the situation in many other countries studied to date, where *C. parvum* tends to be the major agent detected [12]. Although this difference is challenging to explain, it might relate to different farming practices or simply that *C. parvum* is not widely established in livestock populations in some regions of China. Nonetheless, there may be seasonal differences in the presence of *C. bovis* and *C. parvum*. Indeed, Wang et al. [21] proposed that a seasonal shift might be responsible for the dominance of one or more *Cryptosporidium* species over others in pre-weaned calves. Future, large-scale temporal and spatial studies are required to test this hypothesis.

As seen in this study, *C. andersoni*, *C. bovis* and *C. ryanae* infections predominated in post-weaned calves, and are normally not associated with obvious clinical signs in the cattle [20, 50–52]. Nevertheless, such subclinical *Cryptosporidium* infections in cattle should not be neglected, as they may relate to chronic infections and can lead to decreased feed efficiency [53], impaired weight gain or weight loss [54] and milk production losses in dairy cows [52]. Given the expansion of the dairy industry in Hubei Province in China, further studies might explore the epidemiological significance of *Cryptosporidium* genotypes and the sub-structuring of *Cryptosporidium* populations in dairy cattle.

### *Giardia*

*Giardia* DNA was detected in 77 faecal DNA samples. Similar to the results for *Cryptosporidium*, *G. duodenalis* was also found in calves on all six farms, with an average prevalence of 22.6% (Table 1). This number was consistent with previous studies of pre- and post-weaned calves from other cities/provinces of China [25, 55] as well as other countries (cf. [12]). The prevalence of *Giardia* varied from farm to farm in Hubei Province. Farm Chezhan had the highest percentage (60.0%; 12 of 20) of infected calves, whereas farm Yangzijiang had the lowest percentage (9.7%; 9 of 93). There was no significant difference in the prevalence of *G. duodenalis* between the beef and dairy farms (*χ* ^2^ = 0.007, *df* = 1, *P* = 0.933).

The age and gender distributions of *G. duodenalis* are shown in Table 2. *Giardia* was found across both sexes and all ages of calves (Table 2). The prevalences of *G. duodenalis* were 18.5% (49 of 265) and 37.8% (28 of 74) in pre- and post-weaned calves, respectively, with a significant difference (*χ* ^2^ = 12.335, *df* = 1, *P* = 0.0004) between the two groups. According to gender, the results showed that 22.4% (11 of 49) of males and 22.8% (66 of 290) of females were test-positive for *Giardia* (Table 2). Nevertheless, there was no significant difference between the sexes (*χ* ^2^ = 0.002, *df* = 1, *P* = 0.964).

The sequencing of amplicons from *Giardia*-positive samples (*n* = 77) revealed assemblage E. This assemblage has been commonly detected in previous studies of pre- and post-weaned calves from other regions in China, including Heilongjiang [23, 56], Shaanxi [25], Beijing [17], Henan [24], Liaoning [23], Jinling [23] and Shanghai [26], as well as other countries in the world [12]. The p*SSUg* sequences (292 bp) determined here were the same as those representing *G. duodenalis* assemblage E isolates from cattle from China (GenBank: KF843921 [24]), Brazil (GenBank: JF957620; unpublished data) and the USA (GenBank: JN375981 [57]); (GenBank: AY655701 [58]), as well as from deer (GenBank: KX259145; unpublished data) and takin (*Budorcas taxicolor*) (GenBank: KR048491 [59]) from China. Although current preliminary results of *G. duodenalis* from calves in Hubei Province suggest that the public health risk of zoonotic giardiasis is low here, previous longitudinal investigations of cattle giardiasis have indicated the possibility of the transient presence of zoonotic assemblages [60]. Although assemblage E, identified in the present study, had not been considered zoonotic [3, 4], some recent studies describe the occurrence of assemblage E in humans in Queensland, Australia [61], Brazil [62] and Egypt [63–65], indicating that this assemblage can indeed be zoonotic. These recent findings could have important public implications in rural regions of countries such as China, where there is a close relationship between livestock and humans. Therefore, large-scale temporal and spatial studies should be conducted in the future to assess the molecular epidemiology and zoonotic potential of *G. duodenalis* assemblages in different age groups and breeds of cattle and in humans in agricultural regions of Hubei Province.

## Conclusions

In the present study, for the first time, we have reported the prevalence of *Cryptosporidium* and *Giardia* in pre- and post-weaned calves in Hubei Province, China. Based on this ‘snap shot’ study, the prevalence of *G. duodenalis* was shown to be higher than that of *Cryptosporidium* spp. in calves in this province. *Cryptosporidium bovis* was identified as the predominant species in pre-weaned calves, consistent with findings from previous studies from other regions in China. In order to better understand the transmission of these enteric pathogens in China, further work is needed to evaluate the prevalence in large numbers of cattle from different age groups and breeds in different regions of China and at different time points throughout the year, and also to gain relevant information regarding *Cryptosporidium* and *Giardia* in humans and other animals in one of the fastest growing agricultural and economic regions in China (Hubei).

## Additional files

Additional file 1: Table S1 Oligonucleotide primers and PCR protocol used in this study. (XLSX 37 kb)

Additional file 2: Table S2 Summary of information on the reference sequences from the GenBank database used in the present study. (XLSX 55 kb)

## Abbreviations

p*LSUc*: a portion of the large subunit of the nuclear ribosomal RNA gene of *Cryptosporidium*
p*SSUc*: a portion of the small subunit of nuclear ribosomal RNA genes of *Cryptosporidium*
p*SSUg*: a portion of the small subunit of nuclear ribosomal RNA genes of *Giardia*
